# Serum opacity factor normalizes erythrocyte morphology in Scarb1^−/−^ mice in an HDL-free cholesterol-dependent way

**DOI:** 10.1016/j.jlr.2023.100456

**Published:** 2023-10-10

**Authors:** Ziyi Wang, Dedipya Yelamanchili, Jing Liu, Antonio M. Gotto, Corina Rosales, Baiba K. Gillard, Henry J. Pownall

**Affiliations:** 1 Center for Bioenergetics, Houston Methodist, Houston, TX, USA; 2 Departments of Endocrinology and Xiangya Hospital, Central South University, Changsha, China; 3 Departments of Endocrinology and Cardiovascular Medicine, Xiangya Hospital, Central South University, Changsha, China; 4 Department of Medicine, Weill Cornell Medicine, New York, NY, USA

**Keywords:** cholesterol, HDLs, hyperalphalipoproteinemia, scavenger receptor class B member 1, atherosclerosis, erythrocyte morphology

## Abstract

Compared with WT mice, HDL receptor-deficient (Scarb1^−/−^) mice have higher plasma levels of free cholesterol (FC)-rich HDL and exhibit multiple pathologies associated with a high mol% FC in ovaries, platelets, and erythrocytes, which are reversed by lowering HDL. Bacterial serum opacity factor (SOF) catalyzes the opacification of plasma by targeting and quantitatively converting HDL to neo HDL (HDL remnant), a cholesterol ester-rich microemulsion, and lipid-free APOA1. SOF delivery with an adeno-associated virus (AAV_SOF_) constitutively lowers plasma HDL-FC and reverses female infertility in Scarb1^−/−^ mice in an HDL-dependent way. We tested whether AAV_SOF_ delivery to Scarb1^−/−^ mice will normalize erythrocyte morphology in an HDL-FC-dependent way. We determined erythrocyte morphology and FC content (mol%) in three groups—WT, untreated Scarb1^−/−^ (control), and Scarb1^−/−^ mice receiving AAV_SOF_—and correlated these with their respective HDL-mol% FC. Plasma-, HDL-, and tissue-lipid compositions were also determined. Plasma- and HDL-mol% FC positively correlated across all groups. Among Scarb1^−/−^ mice, AAV_SOF_ treatment normalized reticulocyte number, erythrocyte morphology, and erythrocyte-mol% FC. Erythrocyte-mol% FC positively correlated with HDL-mol% FC and with both the number of reticulocytes and abnormal erythrocytes. AAV_SOF_ treatment also reduced FC of extravascular tissues to a lesser extent. HDL-FC spontaneously transfers from plasma HDL to cell membranes. AAV_SOF_ treatment lowers erythrocyte-FC and normalizes erythrocyte morphology and lipid composition by reducing HDL-mol% FC.

## Graphical Abstract

**Figure:**
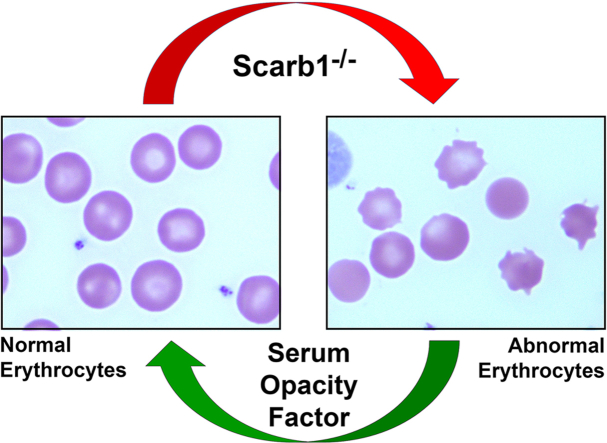

Lipids—free cholesterol (FC), cholesteryl esters (CEs), phospholipids (PLs), and triglycerides (TGs)—are essential components of some cell types and all mammalian plasma lipoproteins. FC is a precursor to steroid hormones, bile acids, and vitamin D. CE and TG, which are produced via the esterification-mediated detoxification of FC and nonesterified fatty acids, form distinct near-homogeneous domains in lipoproteins and lipid droplets within cells. In lipoproteins and the plasma membranes of cells, PLs, the essential FC “solvent,” form surface monolayers and bilayer membranes, respectively, that surround neutral lipids in lipoproteins and the cytoplasm and lipid droplets of living cells. Many human diseases are associated with perturbations in the lipid compositions of cells and plasma lipoproteins, including disorders of lipid metabolism (1) and infectious diseases (2, 3). Plasma and tissue lipid compositions are frequently altered by changes in the expression and structures of proteins associated with lipid metabolism. Deficiencies in lecithin-cholesterol acyltransferase (4), the major FC-esterifying activity in mammalian plasma, the low (5) and HDL receptors (6), and lipolytic enzymes in plasma (7) and peripheral tissue (8) are associated with profound phenotypes (9, 10, 11, 12, 13).

Serum opacity factor (SOF) is a bacterial virulence factor that has an unprecedented activity and mechanism. SOF disrupts HDL structure by releasing lipid-free APOA1 and a small remnant neo HDL, with the concurrent coalescence of nearly all the neutral lipids of >100,000 HDL particles into a CE-rich microemulsion (CERM) that contains APOE and its dimer with APOA2 as its sole apolipoproteins (14, 15). The products of the SOF reaction against HDL support reverse cholesterol transport (i.e., the transfer of peripheral tissue FC to the liver for metabolism (16, 17, 18, 19) and disposal) (20). Infusion of low-dose (4 μg) SOF into WT mice reduces their plasma cholesterol by ∼40% (21).

In mice, SR-B1, the HDL receptor (scavenger receptor class B member 1) is encoded by Scarb1. Mice deficient in this gene (Scarb1^−/−^) have very high plasma concentrations of FC-rich HDL (22, 23, 24, 25), which produce a state of high HDL-FC bioavailability (HDL-FCBI) (25, 26), which is formulated in the Materials and Methods section. FCBI has been described as “active” or accessible cholesterol or, physicochemically as fugacity, an escape tendency analogous to that of the evaporation of a liquid (27, 28, 29, 30, 31, 32). The state of high FCBI, mostly as HDL-FC, among Scarb1^−/−^ mice increases the FC content of some but not all tissues (25). Elevated tissue-FC among Scarb1^−/−^ mice is associated with pathologies in heart (33), the arterial wall (atherosclerosis) (23, 34), erythrocytes (altered morphology) (25, 35, 36), adrenals (37), thymocytes (38), and ovaries, which underlies the infertility observed among female Scarb1^−/−^ mice (39, 40, 41). Tissues spared the effects of excess FC—brain, kidney, and spleen—do not exhibit any overt pathologies. The magnitude of FC transfer from the HDL of Scarb1^−/−^ versus WT mice to macrophages is higher (+300%) likely explaining atherosclerosis in these mice (25).

Given the profound effects of a high plasma HDL-FCBI among Scarb1^−/−^ mice on cellular tissue cholesterol content and the observations that SOF infusion reduces plasma HDL concentrations in mice and its expression rescues fertility in female Scarb1^−/−^ mice (42), we tested the hypothesis that adeno-associated viral delivery of SOF (AAV_SOF_) to Scarb1^−/−^ mice will reduce the constitutively high tissue and cellular FC contents of Scarb1^−/−^ mice and normalize erythrocyte FC content and morphology.

## Materials and Methods

### Formulation of FCBI

Given that FC and its solvent PL are confined to the same compartments in membranes and lipoproteins, we defined HDL-FCBI according to Equation 1 (26).

(1) HDL-FCBI = HDL-P × HDL-mol% FC

where HDL-P is the HDL particle number, and

(2) HDL-mol% FC = 100 × N_FC_/(N_FC_ + N_PL_)

where N_FC_ and N_PL_ are the moles of FC and PL, respectively. According to Equation 1, a high HDL-P and high HDL-mol% FC underlies a high HDL-FC escape tendency, which increases the amount of FC transfer to other sites—tissues and cells.

### Mouse management

All animal studies were approved by the Institutional Animal Use and Care Committee at the Houston Methodist Research Institute. Scarb1^−/−^ and WT C57BL/6J mice (strain no.: 003379 and 000664, respectively; Jackson Laboratory) were maintained on normal laboratory diet (Teklad Envigo; catalog no.: 2920). Mice were periodically genotyped to confirm genetic fidelity; expression of the targeted and WT Scarb1 alleles was confirmed by PCR amplification of DNA extracted from ear punches (primers 5′-GAT-GGG-ACA-TGG-GAC-ACG-AAG-CCA-TTCT-3′ and 5′-TCT-GTC-TCC-GTC-TCC-TTC-AGG-TCC-TGA-3′). All studies were conducted in male and female mice at 12–25 weeks of age except those analyzed for adrenal lipid compositions, which were 8–40 weeks old. Numbers of mice used for the various analyses are given in the legends to figures. We correlated plasma, tissue, and erythrocyte lipid compositions and erythrocyte morphology in three groups of male and female mice—WT, Scarb1^−/−^, and Scarb1^−/−^ mice receiving AAV_SOF_.

### AAV_SOF_ treatment

Recombinant SOF, an 80 kDa truncated protein containing full opacification activity, was expressed and isolated from a bacterial expression system as previously described (14, 15). The development of an AAV_SOF_ and its use for treatment of mice has also been described (42). AAV_SOF_ markedly reduces plasma total cholesterol (TC) and HDL-C levels, whereas the control plasmid AAV_GFP_ does not (42). Male and female Scarb1^−/−^ mice aged 12–13 weeks were treated with AAV_SOF_ by intraperitoneal injection at the rate of 1.2 × 10^11^ genome copies/mouse. Mice were euthanized 3 weeks after AAV_SOF_ injection.

### Tissue lipid extraction

Mice were euthanized, and their blood was collected by heart puncture into EDTA; tissues were perfused with PBS and harvested for lipid and protein analyses (18, 21, 43). Tissues were weighed, homogenized, and extracted (hexane:2-propanol:acetic acid = 3:2:1% v/v/v) (43). Tissue-protein was solubilized with 0.4 M NaOH + 1% sodium dodecyl sulfate. Extracted lipids were dissolved in 1% Triton in chloroform, the chloroform was evaporated under nitrogen, and the lipids were solubilized in water for analysis. Compositions were expressed as lipid mass/protein mass. Previously published compositional data for WT and Scarb1^−/−^ mice (25) are included with additional WT and Scarb1^−/−^ mice data and data from the AAV_SOF_-treated mice.

### Lipoprotein isolation

Lipoproteins were isolated from pooled mouse plasma (5–10/genotype) by sequential flotation (44, 45). Purity was verified by size-exclusion chromatography (20) and compositional analyses. HDL from individual mice was isolated by heparin-manganese precipitation of plasma APOB lipoproteins (46, 47). Plasma and tissue lipids were determined using enzyme-based assays for FC, TC, PL, and TG (Fujifilm Wako Diagnostics, Inc). Cholesteryl ester (CE) concentrations were calculated as (mg TC − mg FC) × 1.6. When TC and FC are essentially equal, a small experimental error in the quantitative assay for TC or FC can result in a negative value for the CE and a ratio FC/TC >1.0. We show the data as calculated and did not set all negative CE values as zero values in order to obtain a valid standard deviation for the dataset. Protein was determined by the DC Protein Assay (Bio-Rad, Inc).

### Erythrocyte analysis

Blood was collected into EDTA by heart puncture. For lipid analysis, blood was centrifuged to sediment erythrocytes, which were washed and collected, and extracted for lipids as described above. Aliquots of whole blood were used to prepare blood smears for morphological analysis according to the vendor protocol (Sigma Aldrich; catalog no.: 620-75) as follows: blood (1–3 μl) was smeared onto a clean microscope slide, air dried, and fixed with absolute methanol for 2 min. Slides were immersed in Wright Stain (catalog no.: 740) for 4 min and modified Giemsa stain (catalog no.: 620) for 8 min, rinsed two times, 1 min each, air dried at room temperature, and fixed with a drop of HistoChoice Mounting Media (Amresco; catalog no.: H157) under a cover slip. Slides were examined under a microscope (100× oil immersion objective), and individual cells, classified as reticulocytes, normal erythrocytes, or abnormal erythrocytes (acanthocytes), were counted. Calculated percent abnormal cells and reticulocytes are the average scores of two blinded observers who viewed the same slides.

### Statistical analysis

Data are presented in the figures as individual values, with mean ± SD, in bar graphs (Sigma Plot 12, Systat Software, Inc.) and correlation plots (Prism 9). Group means were compared by one-way ANOVA with Tukey comparison of means (Prism 9, GraphPad Software, LLC). Linear regression analyses were done using Prism 9. Differences in the plasma, lipoprotein, and tissue lipid compositions of WT versus Scarb1^−/−^ mice with and without treatment with AAV_SOF_ were identified by Tukey comparison of means. Because our previous work (25) showed that the lipid compositions of plasma, plasma lipoproteins, and multiple tissue sites of WT and Scarb1^−/−^ mice differed between sexes for some tissues (25), male and female mice were analyzed separately. For comparisons of genotypes and treatment, the bar graphs show the statistical *P* values within the same sex, that is, WT-female versus Scarb1^−/−^ female and Scarb1^−/−^ female treated with AAV_SOF_, and WT-male versus Scarb1^−/−^ male and Scarb1^−/−^ male treated with AAV_SOF_. Previous WT and Scarb1^−/−^ data have been included for comparison with our new data on WT, Scarb1^−/−^, and Scarb1^−/−^AAV_SOF_-treated mice. Differences between males and females of the same genotype or treatment are given in the figure legends when significant (*P* < 0.05).

## Results

### Plasma and HDL lipids

**Fig. 1:**
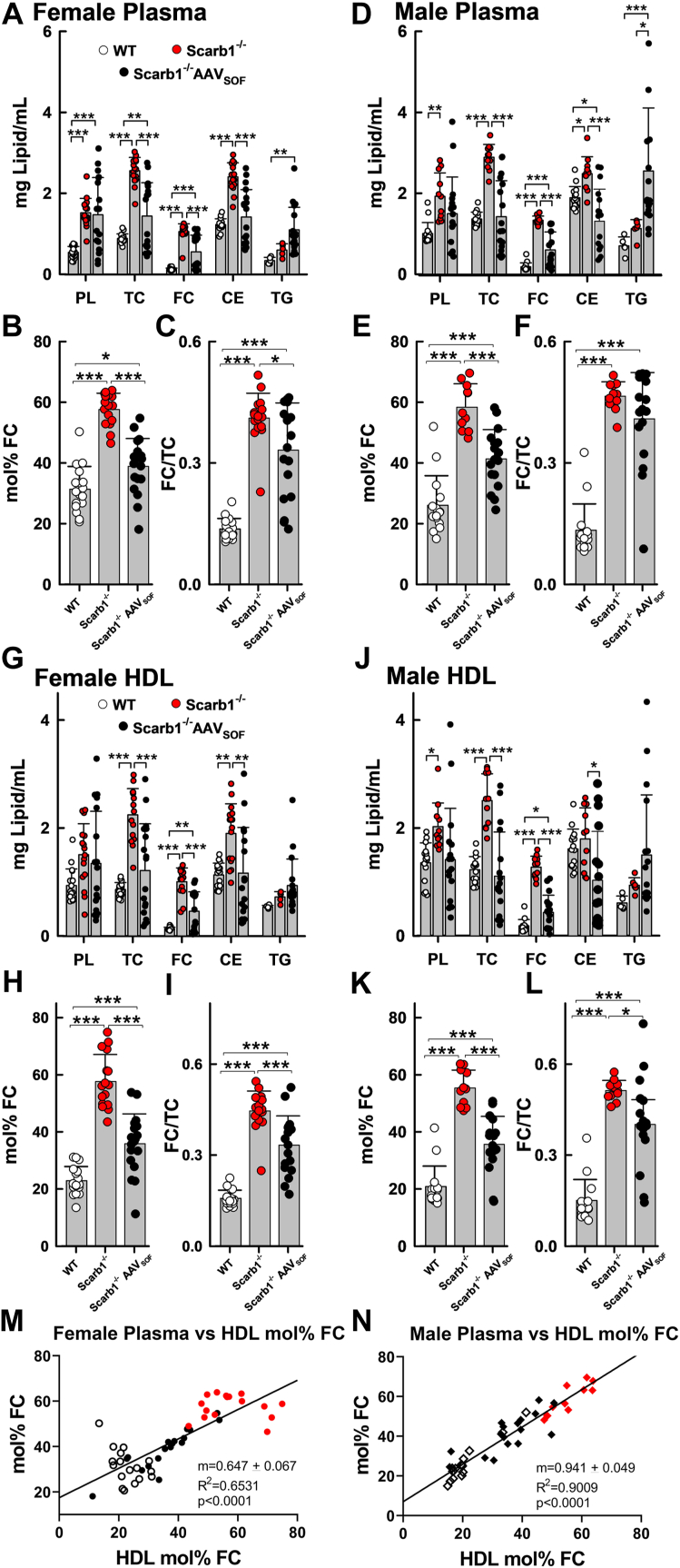
Plasma and HDL lipid composition. AAV_SOF_ treatment decreases the elevated plasma and HDL TC, FC, CE, mol% FC, and FC/TC levels in Scarb1^−/−^ mice toward WT levels. Plasma (A–F) and HDL (G–L) lipid concentrations of female (left panels) and male (right panels) WT, Scarb1^−/−^, and AAV_SOF_-treated Scarb1^−/−^ mice. M and N: Plasma and HDL-mol% FC were highly correlated for both females (M) and males (N). HDLs were obtained from individual mouse plasma by heparin-manganese depletion of APOB lipoproteins. Data points are values for individual mice, and bars are mean ± SD. Numbers of female (-F) and male (-M) mice per group were WT-F (n = 17), Scarb1^−/−^F (n = 16), Scarb1^−/−^F_AAVSOF_ (n = 18), WT-M (n = 15), Scarb1^−/−^M (n = 11), and Scarb1^−/−^M_AAVSOF_ (n = 17). Group means were compared by ANOVA with Tukey comparison of means as described in the Materials and Methods section. *P* values for significantly different pairwise comparisons (∗*P* ≤ 0.05, ∗∗*P* ≤ 0.01, and∗∗∗*P* ≤ 0.001) are indicated over brackets. The slope m, *R*^2^, and *P* values for the linear regression line for the correlation plots (M, N) are shown on the graphs. Comparisons between male and female data within the same genotype or treatment group for plasma: CE: WT-F < WT-M, *P* = 0.0048; TG: AAV_SOF_-F < AAV_SOF_-M, *P* = 0.0005, whereas for HDL, there were no significant differences between sexes for any of the analytes.

Plasma- and HDL-TC, FC, and CE were higher among both male and female Scarb1^−/−^ versus WT mice but reduced in Scarb1^−/−^ mice receiving AAV_SOF_ (Fig. 1A, D, G, J). Plasma- and HDL-PL were less affected by genotype and SOF treatment. In contrast, HDL-TG was not different among the three genotypes but, notably, plasma TG concentrations among Scarb1^−/−^ mice receiving AAV_SOF_ were higher than those WT and untreated Scarb1^−/−^ mice (males only). Both the mol% FC and the FC/TC were higher in the Scarb1^−/−^ versus WT mice but reduced in the former by AAV_SOF_ delivery (Fig. 1B, C, E, F, H, I, K, L). Plasma- and HDL-mol% FC highly correlated and increased as WT ∼ Scarb1^−/−^AAV_SOF_ < Scarb1^−/−^ (Fig. 1M, N). Plasma- and HDL-FC also correlated and increased similarly (supplemental Fig. S1). The strength of these correlations is reflected in the slopes (m) of the curves, which for HDL-mol% FC versus plasma-mol% FC are 0.65 and 0.94 for females and males, respectively, at or near unity, with *P* < 0.0001 for both.

### Erythrocyte lipids

**Fig. 2:**
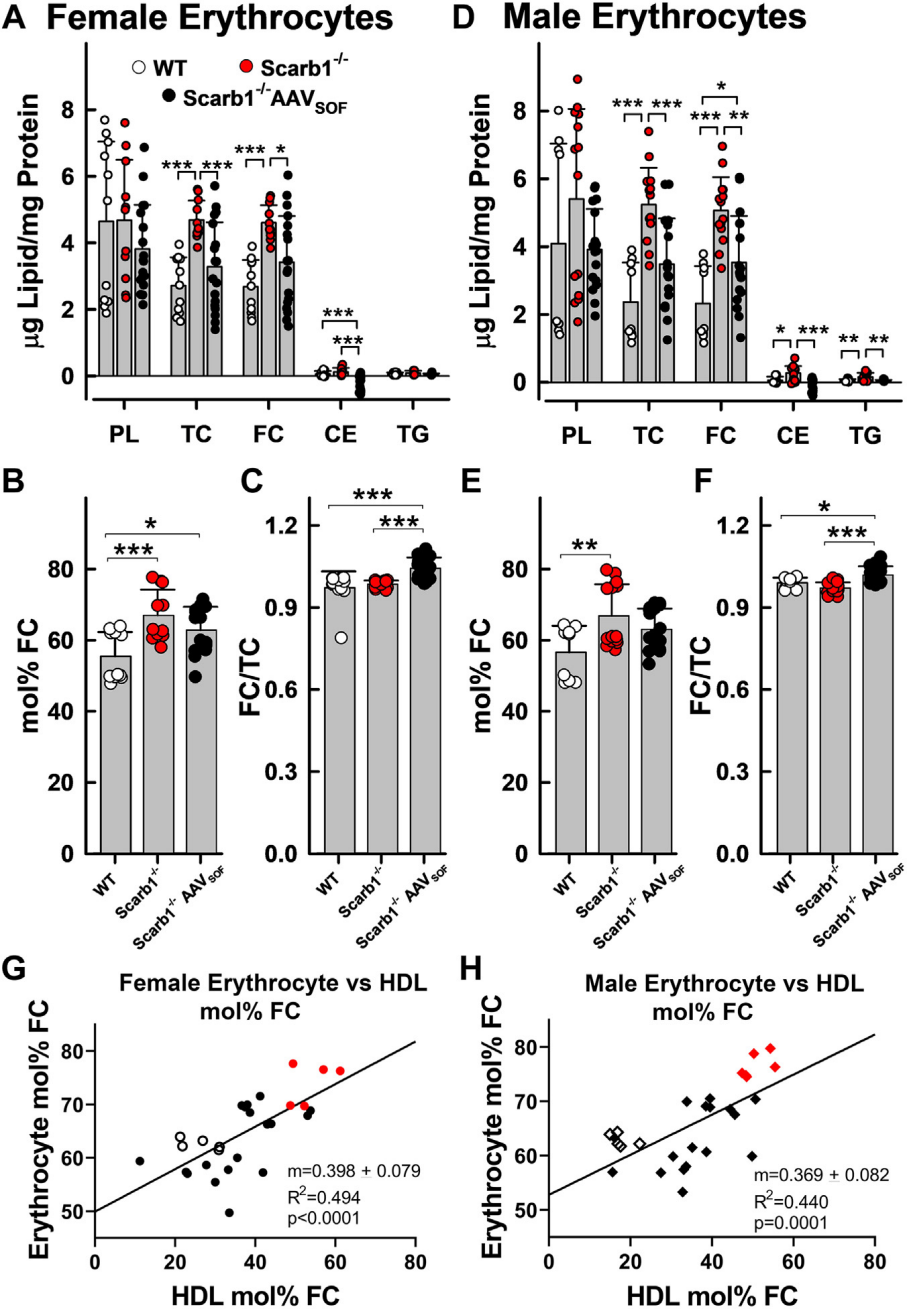
Erythrocyte lipid composition. AAV_SOF_ treatment decreases the elevated erythrocyte (red blood cell) TC, FC, and mol% FC in Scarb1^−/−^ mice toward WT levels. Panels A–C (female) and D–F (male) provide the lipid composition relative to protein (W/W), mol% FC, and the FC/TC ratio (W/W). G and H: Correlation of erythrocyte versus HDL-mol% FC. Data points are values for individual mice, and bars are mean ± SD. Mice/group for the bar graphs were WT-F (n = 11), Scarb1^−/−^F (n = 11), Scarb1^−/−^F_AAVSOF_ (n = 18), WT-M (n = 9), Scarb1^−/−^M (n = 14), and Scarb1^−/−^M_AAVSOF_ (n = 17) and for the correlation plots WT-F (n = 5), Scarb1^−/−^F (n = 5), Scarb1^−/−^F_AAVSOF_ (n = 18), WT-M (n = 5), Scarb1^−/−^M (n = 6), and Scarb1^−/−^M_AAVSOF_ (n = 17). Statistics are as described in the legend to Figure 1. Comparisons between male and female data within the same genotype or treatment group showed no significant differences between sexes for any of the analytes. Note: Data in the correlation plots are only for those mice from which both plasma and erythrocytes were collected so there are fewer paired values for the correlations (G, H) than the total number of mice in the bar graphs.

The lipid compositions of blood erythrocytes from male and female WT versus Scarb1^−/−^ mice were different (Fig. 2A, D). As previously reported (25, 36), the erythrocytes from Scarb1^−/−^ mice were more FC rich than those from WT mice. However, we observed that AAV_SOF_ delivery nearly normalized the erythrocyte-FC contents of both male and female Scarb1^−/−^ mice to near WT values. In spite of the SOF-mediated changes in erythrocyte-FC, erythrocyte-mol% FC (Fig. 2B, E) was not significantly reduced versus Scarb1^−/−^ because of parallel changes in PL and FC contents. Meaningful amounts of CE were not detected in the erythrocytes in any of the mice, so the FC/TC ratios among all mouse erythrocytes were nearly equal to one (Fig. 2C, F). Among both male and female WT mice and Scarb1^−/−^ mice ± AAV_SOF_, HDL-FC content and HDL-mol% FC correlated positively with erythrocyte FC content (Fig. 2G, H). The strength of the correlation is reflected in the slopes of the curves, which are ∼0.4 for erythrocyte-mol% FC versus HDL-mol% FC, *P* < 0.0001 and >2 for erythrocyte-FC versus HDL-FC, *P* < 0.001 (supplemental Fig. S2).

### Erythrocyte morphology

**Fig. 3:**
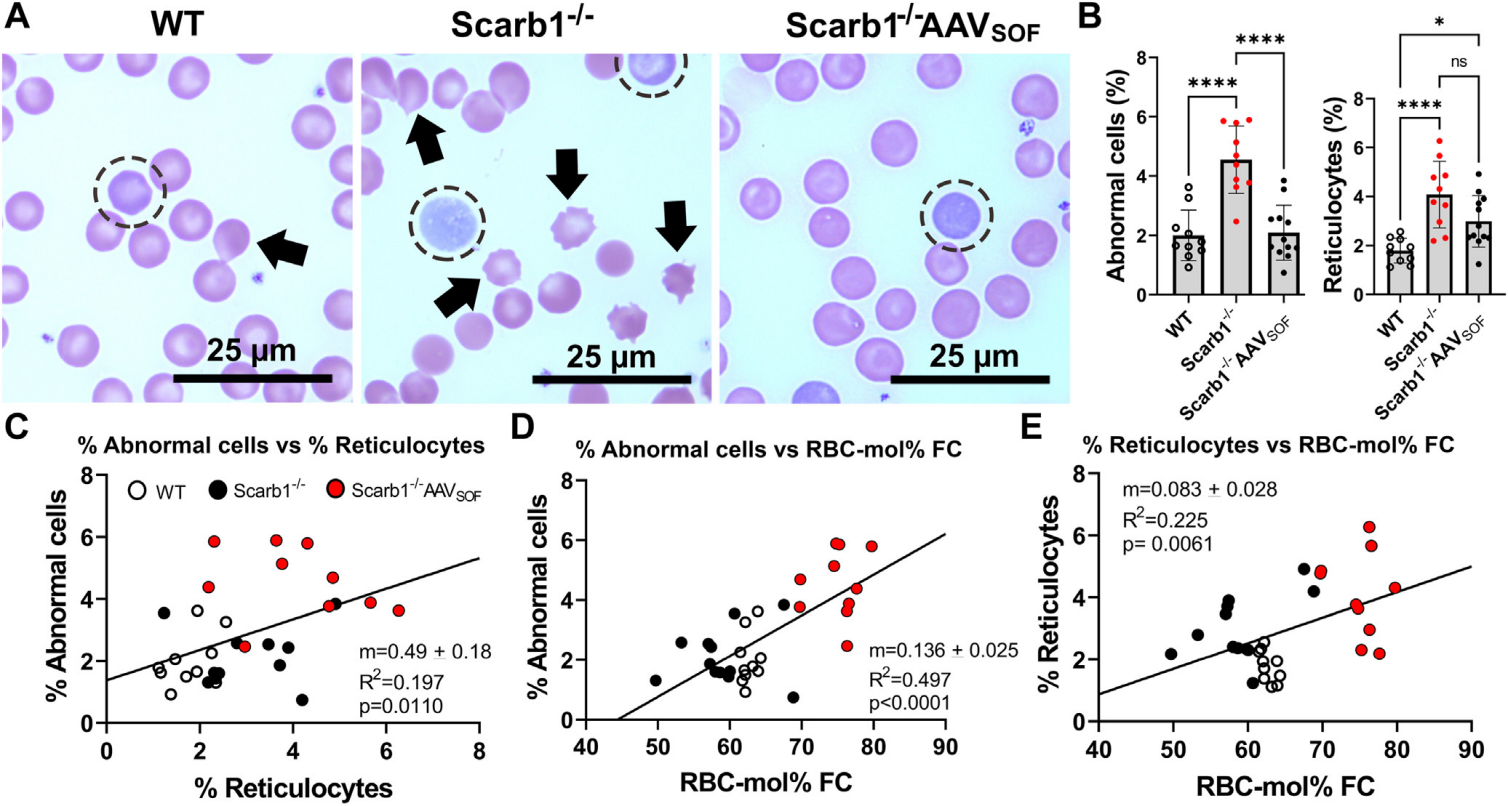
Erythrocyte (red blood cell [RBC]) morphology correlates with RBC-FC. AAV_SOF_ normalizes both the RBC-FC content and morphology. A: Micrographs of representative fields of blood smears stained with Wright/Giemsa stain from WT, Scarb1^−/−^, and AAV_SOF_-treated Scarb1^−/−^ mice (Arrows: abnormal cells; circles: reticulocytes). B, quantitation of erythrocyte morphology based on counts of abnormal cells (acanthocytes) and reticulocytes. Data from male and female mice were not significantly different; so pooled data are shown. C: Correlation of percent abnormal cells versus percent reticulocytes. D and E: Correlation of percent abnormal and percent reticulocyte cells with erythrocyte-mol% FC. Mice/group: WT (n = 10), Scarb1^−/−^ (n = 10), and Scarb1^−/−^_AAVSOF_ (n = 12). Data plotted by sex were not different; so male and female data were pooled.

Given that Scarb1 deletion increases erythrocyte-FC and mol% FC with an attendant disruption of erythrocyte morphology (34, 35, 36), we tested whether AAV_SOF_, which normalizes erythrocyte-FC composition, would also normalize erythrocyte morphology according to the number of abnormal cells and reticulocytes observed. Normal mouse reticulocyte counts are ∼4%, and the abnormal cell count is a bit lower (36). We found that Scarb1 deletion increased the number of abnormal cells and reticulocytes over the normal range, and that this effect was reversed by delivering AAV_SOF_ to the Scarb1^−/−^ mice (Fig. 3A, B). The changes in the number of abnormal cells and reticulocytes induced by Scarb1 ablation and treatment with AAV_SOF_ were positively correlated (Fig. 3C). Moreover, as expected, erythrocyte morphology correlated with mol% FC (Fig. 3D, E) and erythrocyte FC (supplemental Fig. S3).

### Tissue lipids

**Fig. 4:**
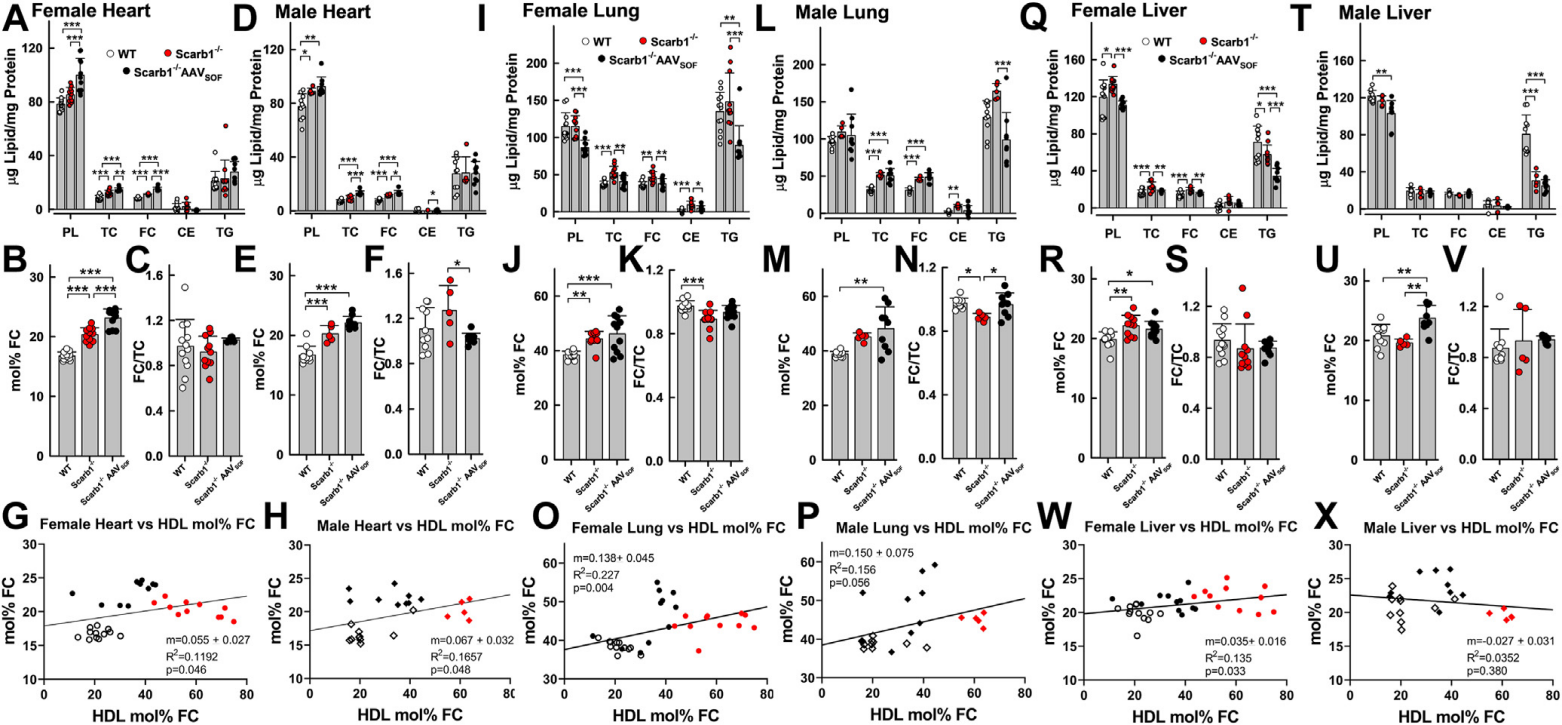
Heart, lung, and liver lipid composition. AAV_SOF_ treatment has variable effects on the cholesterol content of heart, lung, and liver in Scarb1^−/−^ mice. A–H: Heart; (I–P) Lung; and (Q–X) Liver. The respective panels provide the lipid composition relative to protein (W/W), mol% FC, and the FC/TC ratio (W/W). Data points are values for individual mice, and bars are mean ± SD. Mice/group were WT-F (n = 12), Scarb1^−/−^F (n = 11), Scarb1^−/−^F_AAVSOF_ (n = 11), WT-M (n = 10), Scarb1^−/−^M (n = 5), and Scarb1^−/−^M_AAVSOF_ (n = 9). Statistics are as described in the legend to Figure 1. Comparisons between male and female data within the same genotype or treatment group gave the following significant differences between sexes: heart: TC: Scarb1^−/−^F> Scarb1^−/−^M, *P* = 0.0137; FC: AAV_SOF_-F> AAV_SOF_-M, *P* = 0.0084; CE: Scarb1^−/−^F> Scarb1^−/−^M, *P* = 0.0015; FC/TC: Scarb1^−/−^M> Scarb1^−/−^F, *P* = 0.0025. Lung: TC: AAV_SOF_-M> AAV_SOF_-F, *P* = 0.0286; AAV_SOF_-M> AAV_SOF_-F, *P* = 0.0019. Liver: TC: Scarb1^−/−^F> Scarb1^−/−^M, *P* = 0.0085; FC: Scarb1^−/−^F> Scarb1^−/−^M, *P* = 0.0017; TG: Scarb1^−/−^F> Scarb1^−/−^M, *P* = 0.0074; mol% FC: Scarb1^−/−^F> Scarb1^−/−^M, *P* = 0.0587 and AAV_SOF_-M> AAV_SOF_-F, *P* = 0.0470. Note: In some instances, the calculated FC/TC ratio was >1 because of the imprecision of some of the analyses at concentrations near the detection limits of the assay.

The effects of AAV_SOF_ delivery on the FC content of other tissue sites were less profound than those observed in erythrocytes and sometimes varied according to sex. FC is elevated in both male and female heart tissue in Scarb1^−/−^ mice, and AAV_SOF_ treatment increased heart-FC and mol% FC in both sexes. However, AAV_SOF_ reduced the FC/TC ratio in males only (Fig. 4A–F). Nevertheless, heart-mol% FC significantly and positively correlated with HDL-mol% FC in both males and females (Fig. 4G, H). FC is elevated in lungs of both female and male Scarb1^−/−^ mice (Fig. 4I–P). AAV_SOF_ treatment reduced FC in female lungs but not male lungs. AAV_SOF_ does not reduce elevated mol% FC in either male Scarb1^−/−^ or female Scarb1^−/−^ lungs. The effects of AAV_SOF_ in lung were small, but still lung-mol% FC significantly and positively correlated with HDL-mol% FC (Fig. 4O, P). In liver, AAV_SOF_ normalized the elevated FC content and mol% FC in females but increased the mol% FC in males (Fig. 4Q–S, T–V). AAV_SOF_ failed to reverse the effects of Scarb1 deletion on liver-TG and reduced TG content (Fig. 4Q, T). Female but not male liver-mol% FC correlated with HDL mol% FC (Fig. 4W, X).

**Fig. 5:**
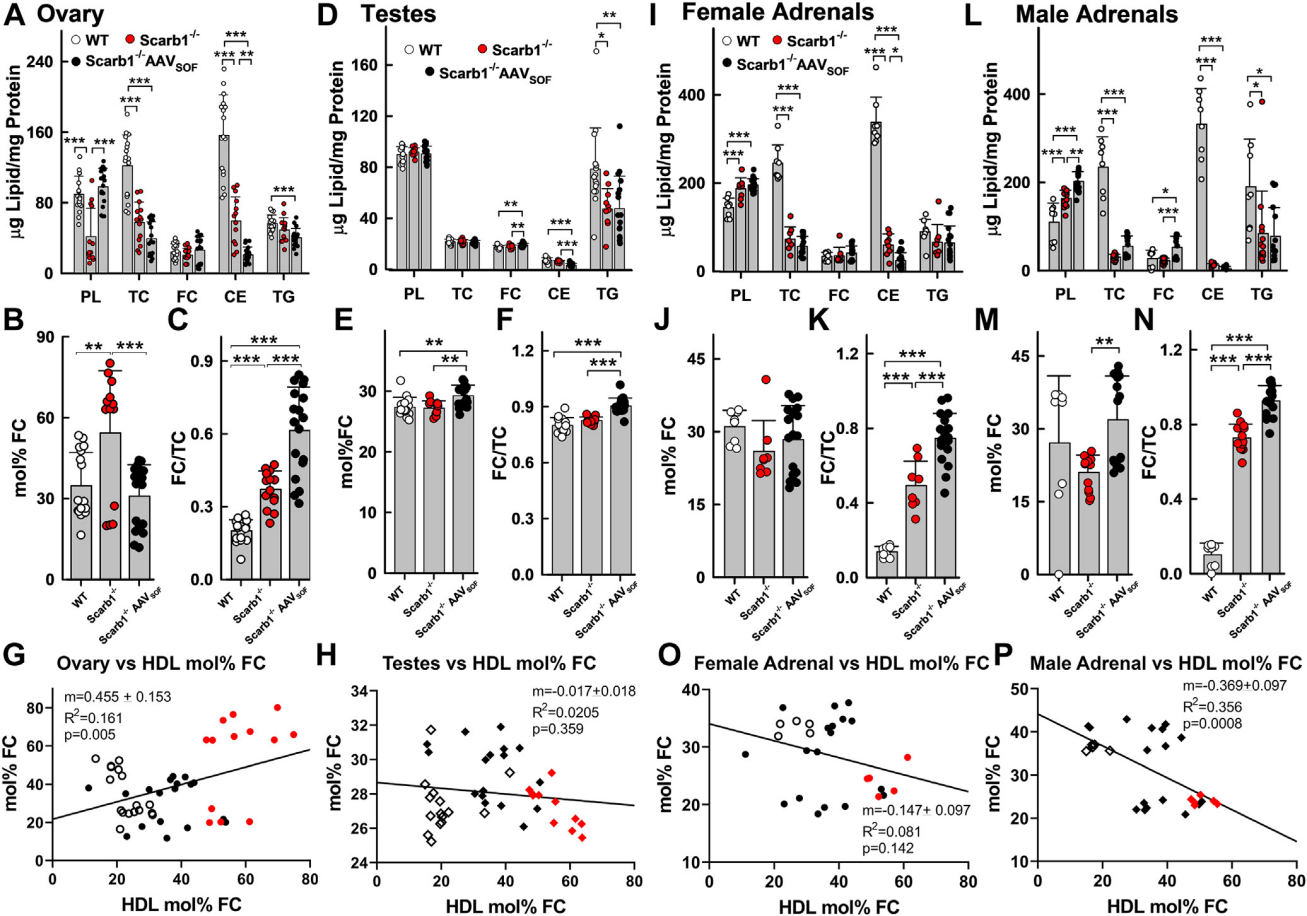
Steroidogenic tissue lipid composition. AAV_SOF_ treatment decreases the elevated mol% FC to WT levels in ovaries but does not restore the low CE levels of ovaries or adrenals of Scarb1^−/−^ mice. A–C and G: ovary; D–F and H: testis, and I–P: adrenals. The respective panels provide the lipid composition relative to protein (W/W), mol% FC, and the FC/TC ratio (W/W). Data points are values for individual mice, and bars are mean ± SD. Mice/group for ovaries : WT-F (n = 17), Scarb1^−/−^F (n = 14), Scarb1^−/−^F_AAVSOF_ (n = 18); for testis, WT-M (n = 15), Scarb1^−/−^M (n = 11), and Scarb1^−/−^M_AAVSOF_ (n = 17); for adrenals: WT-F (n = 5–8), Scarb1^−/−^F (n = 5–8), Scarb1^−/−^F_AAVSOF_ (n = 18), WT-M (n = 5–8), Scarb1^−/−^M (n = 6–13), and Scarb1^−/−^M_AAVSOF_ (n = 17). Statistics are as described in the legend to Figure 1. Comparisons between male and female adrenal data within the same genotype or treatment group gave the following significant differences between sexes: CE: Scarb1^−/−^F> Scarb1^−/−^M, *P* = 0.0426; TG: WT-M> WT-F, *P* = 0.0473; FC/TC: Scarb1^−/−^M> Scarb1^−/−^F, *P* < 0.0001 and AAV_SOF_-M> AAV_SOF_-F, *P* < 0.0001.

Within the steroidogenic tissues, there were some notable effects of AAV_SOF_. In ovaries, AAV_SOF_ reduced CE content, did not change the FC content, but normalized the PL content, thereby normalizing the mol% FC (Fig. 5A, B and Equation 2) but not the FC/TC ratio (Fig. 5C). Ovary-mol% FC correlated with that of HDL-mol% FC (Fig. 5G). Although AAV_SOF_ altered FC and CE content, the mol% FC, and the FC/TC ratio in testes, the effects were small (Fig. 5E–H). Although testes-mol% FC correlated negatively with that of HDL-mol% FC, the effect was also small and not significant. Adrenal TC and CE are reduced in both male and female Scarb1^−/−^ mice. (Fig. 5I–N). The effects of AAV_SOF_ on adrenal lipid composition were small; AAV_SOF_ normalized the mol% FC in males and females (trend) and increased the FC/TC ratio but failed to normalize the profound reduction in CE induced by Scarb1 deletion. Unexpectedly, adrenal-mol% FC correlated negatively with HDL-mol% FC for males (*P* = 0.008) and females (nonsignificant, *P* = 0.142).

In brain, AAV_SOF_ increased FC content, mol% FC, and the FC/TC ratio but reduced CE content; there was no correlation between brain-mol% FC and HDL-mol% FC (supplemental Fig. S4). The effects of AAV_SOF_ on the lipids in the kidney (supplemental Fig. S5), spleen (supplemental Fig. S6), and testes- and ovary-fat (supplemental Fig. S7) were mostly small and not significant except for the paradoxical increase in FC, mol% FC, and TG (>+300%) in testes- and ovary-fat (supplemental Fig. S7A–F). In spleen, the major effect was a reduction in TG content, whereas in kidneys, AAV_SOF_ increased FC content and mol% FC to WT levels only in males. In kidney, spleen, and fat tissues, tissue-mol% FC and HDL-mol% FC were not correlated.

### Sex differences in tissue-lipids and response to AAV_SOF_

In our previous study of tissue lipids in the Scarb1^−/−^ versus WT mouse (25), we found significant differences between lipid content of males versus females of the same genotype in some of the tissues. In this study, male and female data are presented in separate panels, and male versus female differences are summarized in the figure legends. Response of tissue lipids to AAV_SOF_ treatment also varied by sex. For example, FC is higher in lungs of both female and male Scarb1^−/−^ versus WT mice, but AAV_SOF_ treatment reduces FC to WT levels in female lungs but not male lungs (Fig. 4I, L). In liver, FC is elevated in female Scarb1^−/−^ mice and reduced with AAV_SOF_ treatment, whereas FC in liver of male Scarb1^−/−^ does not differ from WT and is not altered with AAV_SOF_ treatment (Fig. 4Q, T). In kidney, FC in Scarb1^−/−^ females does not differ from WT and is not altered by AAV_SOF_, whereas FC in Scarb1^−/−^ male mice is lower than in WT mice, and AAV_SOF_ increases FC to WT levels and increases mol% FC in the male mice (supplemental Fig. S6). In spleen, FC is lower in female Scarb1^−/−^ versus WT mice, AAV_SOF_ decreases FC further, but mol% FC is the same in all three groups of female mice. FC is higher in spleens of male Scarb1^−/−^ mice versus WT mice, AAV_SOF_ reduced spleen FC below WT levels and decreased spleen-mol% FC. The correlations between HDL-mol% FC and FC for erythrocytes and all tissues are summarized in supplemental Table S1.

## Discussion

HDL receptor deficiency in Scarb1^−/−^ mice leads to the accretion of HDL that is dysfunctional because it occurs at a higher plasma concentration and contains more FC than receptor-competent WT HDL. This creates a state of high FCBI, which has been described as “active cholesterol” and fugacity. We use the term bioavailability because of the biological consequences of FC escape from HDL in vivo. HDL-FC is highly mobile and rapidly clears from plasma in mice (*t*_1/2_ = 5 min) (48) and humans (*t*_1/2_ = 9 min) (49) and in mice rapidly transfers to nearly all tissues (48). Whereas FC uptake in some tissues, such as liver, is mediated by SR-B1, transfer to many other tissues, including the intestine, occurs by diffusion, sometimes called transintestinal cholesterol efflux (50). Previous in vitro studies showed that HDL-FC transfer to cells increases with increasing HDL concentration and HDL-FC content (51) so that one would expect more FC to transfer to cells and tissues from HDL of Scarb1^−/−^ versus WT mice. Scarb1^−/−^ mice present with multiple pathologies—female infertility, atherosclerotic cardiovascular disease, and platelet and erythrocyte abnormalities (34, 36, 39, 52). Some of these effects—erythrocyte abnormalities, (34) atherosclerosis (34), and infertility (39)—are reversed by the HDL-lowering drug, probucol, or by AAV_SOF_ delivery (42). In vitro, SOF activity versus HDL produces lipid-free APOA1 and remnant HDL, but in vivo, these rapidly convert to HDL and are hepatically extracted (17), and the plasma HDL concentration declines. Thus, we tested the hypothesis that AAV_SOF_-mediated reduction of plasma- and HDL-FC would also normalize lipid composition and morphology of erythrocytes, which are also FC acceptors in other settings, including cholesterol efflux (53).

While WT and Scarb1^−/−^ mice have similar hematocrits, Scarb1^−/−^ mice have greater erythrocyte volumes and lower erythrocyte hemoglobin, and according to filipin staining, the cholesterol content of erythrocytes from Scarb1^−/−^ mice is higher than that of WT mice (36). We quantified this observation using an enzymatic assay and further showed that plasma-, HDL-, and erythrocyte-mol% FC is highest in Scarb1^−/−^ compared with AAV_SOF_-exposed Scarb1^−/−^ mice and lowest in WT mice. Given that HDL is the major lipoprotein in mice, high HDL-mol% FC underlies the high plasma-mol% FC and is the major source of FC, which in turn drives erythrocyte-mol% FC. The strong correlation is reflected in the slopes of the curves for plasma-mol% FC versus HDL-mol% FC, which are close to unity (Fig. 1M, N). The correlations between erythrocyte-mol% FC versus HDL-mol% FC are equally robust with slopes of 0.40 and 0.37 for females and males, respectively. The slopes are less than unity suggesting that the mol% FC are at steady state but not at equilibrium and that the erythrocyte-mol% FC is less than that of HDL-mol% FC.

FC accretion by erythrocytes is higher than in many other cells or tissues; this occurs for two reasons. First, erythrocytes reside in the same compartment, namely plasma, as the source of excess FC, HDL. Second, erythrocytes have no metabolic defense against a high FCBI; they lack the intracellular FC-esterifying enzymes and plasma membrane lipid transporters that detoxify or exocytose FC, respectively. Thus, under conditions of high FCBI, erythrocytes, like LDLs (25), which is also confined to the plasma compartment, rapidly (*t*_1/2_ = ∼3 min) accumulate FC (i.e., faster than the rate of FC transfer to the major tissues) (48). Changes in HDL-mol% FC have a greater effect on abnormal cell formation versus reticulocyte production: the effects of an increased HDL-mol% FC on abnormal cell formation were greater (m = 0.136) than its effects on reticulocyte production (m = 0.083; Fig. 3D, E).

A high erythrocyte-FC is associated with an increased number of abnormal erythrocytes, characterized by “blebbing.” The erythrocyte-plasma membrane is asymmetric with respect to FC distribution (54, 55, 56, 57, 58, 59, 60). One estimate using orthogonal lipid sensors puts the outer leaflet ratio to inner leaflet ratio at ∼12 (61). Consistently, most studies observed more FC in the outer versus inner leaflet of the plasma membrane even though the ranges of inner-to-outer leaflet FC vary considerably among studies. This occurs despite FC transfer between leaflets on a millisecond time scale (62). This asymmetry has been attributed the asymmetry in the compositions of the FC-binding PLs. The outer leaflet contains more highly cholesterophilic PL—saturated phosphatidylcholines and especially sphingomyelin (63), whereas, the PLs of the inner leaflet are nearly devoid of sphingomyelin and contain ∼80% of the plasma membrane unsaturation (60). As a consequence, under conditions of high FCBI, FC preferentially accumulates in the outer leaflet, and to accommodate the additional FC, blebs form. This structure is maintained because the rates of PL translocation across the plasma membrane are slower than that of FC (62, 64). When the HDL-FC is reduced by either probucol (34) or AAV_SOF_, the normal structures are restored.

FC also transfers rapidly to liver because it is highly perfused (48). Given that probucol, like SOF, reduces HDL-C concentrations, changes in the tissue lipid compositions induced by SOF may also occur in patients receiving probucol and could underlie some of the metabolic consequences of probucol therapy. The elevation of plasma-TG but not HDL-TG by AAV_SOF_ suggests that some products of the SOF reaction, most likely the CERM, compete with either TG hydrolysis, or, more likely, the CERM produced by the SOF competes with endogenous very LDL uptake by the LDL receptor and other lipoprotein receptors (20). This would be consistent with the observation that in most tissues in which TG was altered, that is, lung, liver, ovaries, and male adrenals, kidney, and spleen, TG in the AAV_SOF_ mice was lower than in the Scarb1^−/−^ control or WT mice. The exceptions were fat tissue surrounding the ovaries and testis, in which the TG was elevated in the AAV_SOF_-treated mice (supplemental Fig. S7).

Although underlying mechanisms may differ, many lipid disorders are associated with erythrocyte abnormalities; for example, abetalipoproteinemia (65) is caused by mutations in the gene encoding microsomal TG transfer protein (66). In abetalipoproteinemia, the observed acanthacytosis is associated with the absence of LDL and the occurrence of erythrocytes that are phosphatidylcholine poor and SM rich compared with normolipidemic erythrocytes (67). Compared with a normolipidemic cohort, patients with total deficiency of the FC-esterifying enzyme, lecithin-cholesterol acyltransferase, have a high FC/CE ratio and present with hemolytic anemia, in which there is premature erythrocyte hemolysis that results in anemia. Notably, patients with acute coronary syndrome have higher erythrocyte-FC than do those with stable coronary artery disease, and erythrocyte-FC content better predicted acute coronary syndrome than either HDL-C or C-reactive protein levels (68).

### Limitations of the study

The Scarb1^−/−^ mouse has an extreme phenotype that has not been documented in humans. Thus, the value of the study is mechanistic, revealing how changes in plasma FC concentrations impact tissue FC content, an effect that was shown to be reversible, in part, by delivering AAV_SOF_ to Scarb1^−/−^ mice.

## Conclusions

HDL-FC spontaneously transfers from plasma lipoproteins to cell membranes in multiple tissue sites on a time scale of minutes to a few hours. FC enrichment of erythrocytes, which are in contact with plasma HDL, was more profound than tissue-FC enrichment. AAV_SOF_ treatment lowers both plasma HDL-FC and erythrocyte-FC and normalizes erythrocyte morphology and lipid composition in an HDL-FC-dependent way. Therapy with an SOF mimetic could be useful for treatment of patients who present with high HDL-C bioavailability as a risk factor for atherosclerotic cardiovascular diseases or other metabolic disorders.

## Data Availability

All data are contained within the article.

## Supplemental data

This article contains supplemental data.

## Conflict of interest

The authors declare that they have no conflicts of interest with the contents of this article.
