# Supplementary material for: Serum opacity factor normalizes erythrocyte morphology in Scarb1−/− mice in an HDL-free cholesterol-dependent way
Source: J Lipid Res. 2023 Oct 10;64(11):100456. doi: 10.1016/j.jlr.2023.100456 (PMC10641538; doi:10.1016/j.jlr.2023.100456)
Supplement: Supplemental information [file mmc1.docx]

**Supplemental Information**

**Serum Opacity Factor Normalizes Erythrocyte Morphology in Scarb1^-/-^ Mice in an HDL-Free Cholesterol-Dependent Way**

Ziyi Wang ^1,2^, Dedipya Yelamanchili ^1^, Jing Liu ^1,3^, Antonio M. Gotto Jr.^1,4^, Corina Rosales^1,4^, Baiba K. Gillard^1,4,+^ and Henry J. Pownall ^1,4,+,*^

^1^ Center for Bioenergetics, Houston Methodist, 6565 Fannin Street, Houston TX 77030, USA;

Departments of ^2^ Endocrinology and ^3^ Cardiovascular Medicine, Xiangya Hospital, Central South University, Changsha 410008, China

^4^Department of Medicine, Weill Cornell Medicine, 1300 York Ave, New York, NY, 10065, USA;

**
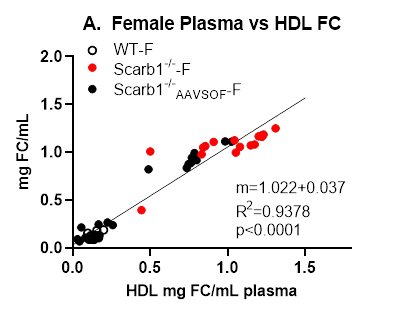
**

**
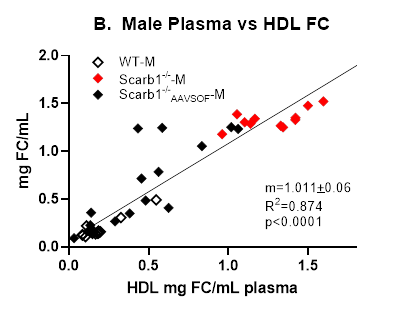
**

**Supplemental Fig. S1.** Plasma- and HDL-FC were highly correlated for both females (**A**) and males (**B**). Statistical analysis as for Figure-1 legend.

**
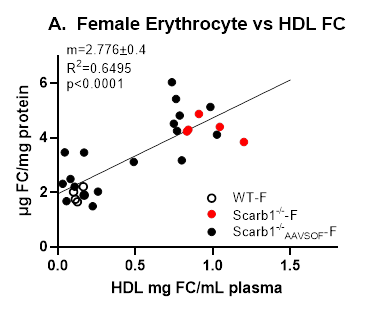
**

**
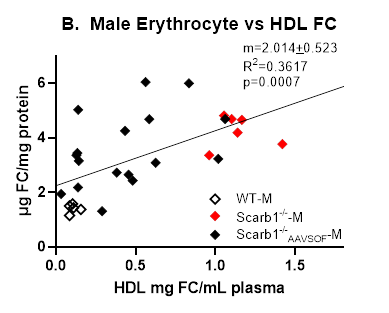
**

**Supplemental Fig. S2.** Correlation of erythrocyte vs HDL-FC for female (**A**) and male (**B**) mice. Statistics are as described in the **Figure-2** legend.

**
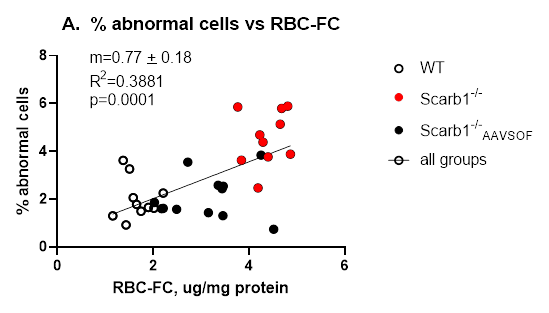
**

**
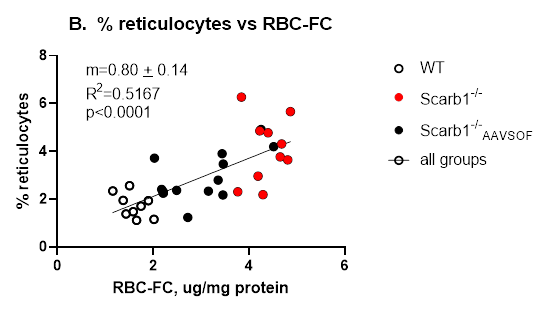
**

**Supplemental Fig. S3.** Correlation of % abnormal cells (**A**) and % reticulocytes(**B**) with erythrocyte-FC. Statistics are as described in the **Figure-3** legend.


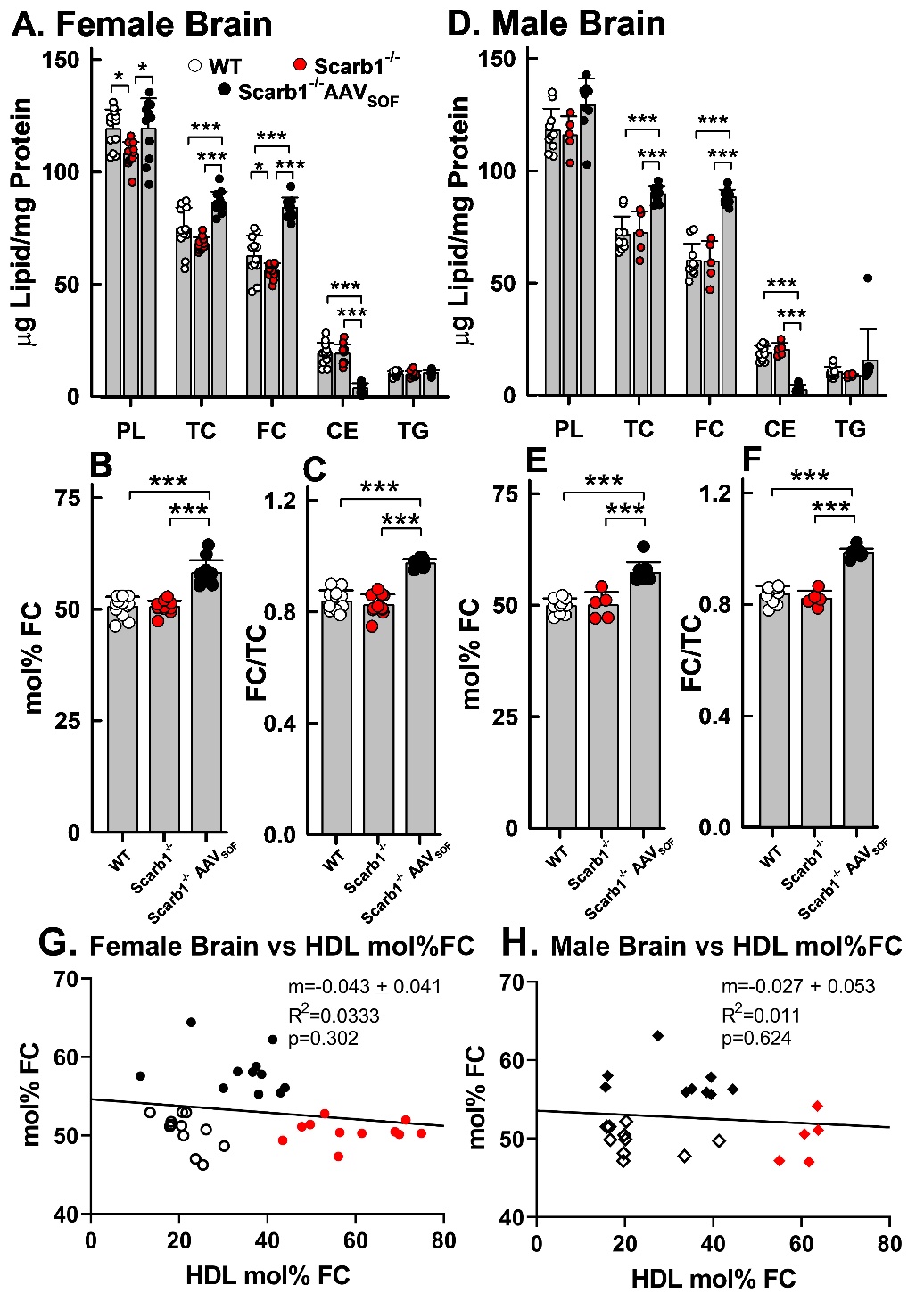


**Supplemental Fig. S4.** Brain lipid composition**.** While Scarb1^-/-^ and WT brain have similar FC and CE content, AAV_SOF_ increases brain FC and decreases brain CE. AAV_SOF_ also increases brain mol % FC and FC/TC. **A-C and G:** Female**. D-F and H:** Male. The respective panels provide the lipid composition relative to protein (W/W), mol% FC, and the FC/TC ratio (W/W). **G, H**: Brain-mol% FC does not correlate with HDL-mol% FC for either female or male mice. Data points are values for individual mice, and bars are mean + SD. Mice/group were WT-F (n = 12), **Scarb1^-/-^**-F (n = 11), **Scarb1^-/-^**-F_AAVSOF_ (n = 11), WT-M (n = 10), **Scarb1^-/-^**-M (n = 5) and **Scarb1^-/-^**-M_AAVSOF_ (n = 9). Comparisons between male and female data within the same genotype or treatment showed no significant differences between male and female values. *P* values for significantly different pairwise comparisons (* *P* < 0.05, ** *P*<0.01, *** *P*<0.001) are indicated over brackets.


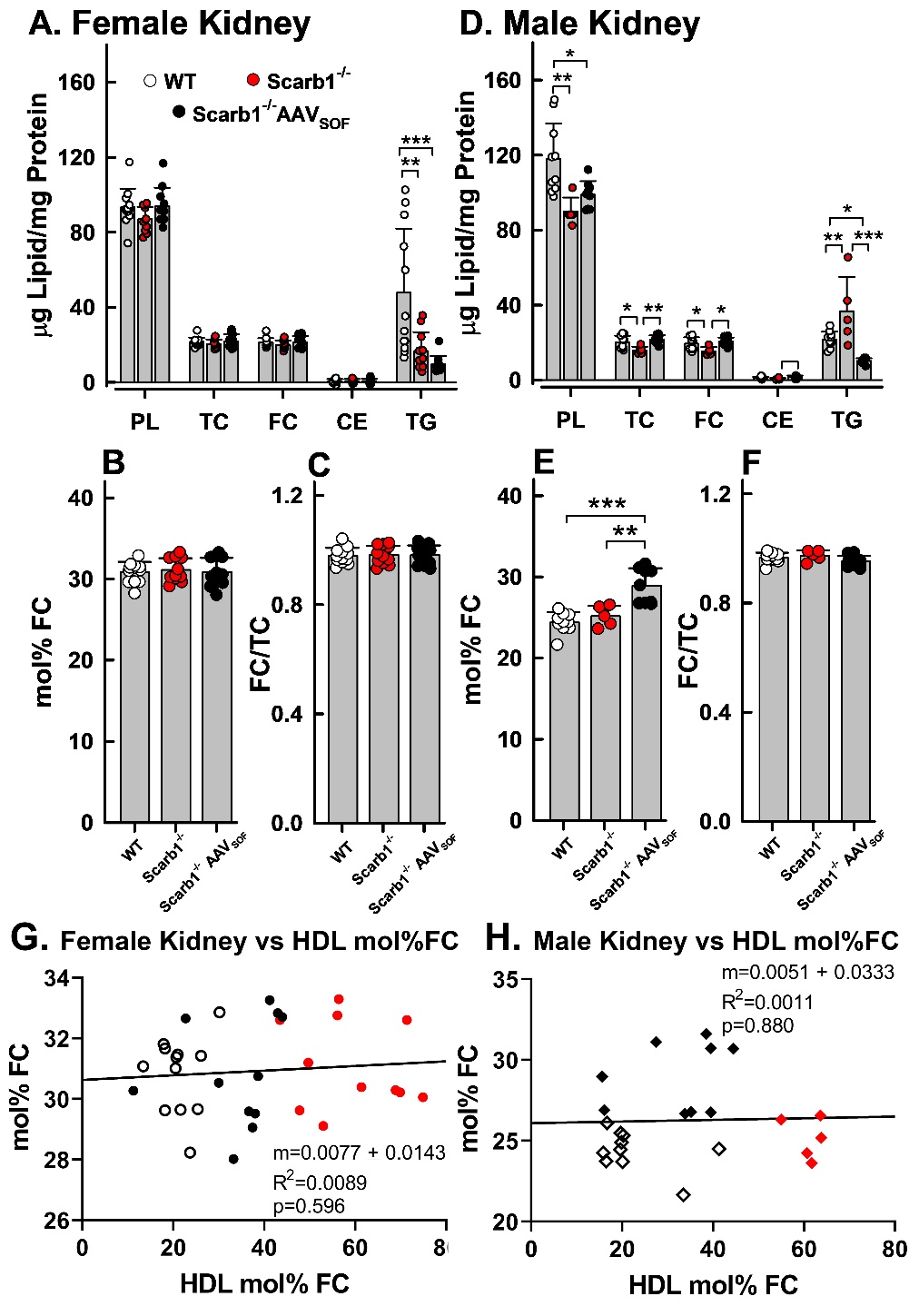


**Supplemental Fig. S5.** Kidney lipid composition**.** FC in Scarb1^-/-^ female mice does not differ from WT, and is not altered by AAV_SOF_. FC in Scarb1^-/-^ male mice is lower than in WT mice, AAV_SOF_ increases FC to WT levels, and increases mol% FC in the male mice. **A-C and G:** Female**. D-F and H:** Male. The respective panels provide the lipid composition relative to protein (W/W), mol% FC, and the FC/TC ratio (W/W). **G, H**: Kidney-mol% FC does not correlate with HDL-mol% FC for either female or male mice. Data points are values for individual mice, and bars are mean + SD. Mice/group were: WT-F (n = 12), **Scarb1^-/-^**-F (n = 11), **Scarb1^-/-^**-F_AAVSOF_ (n = 11), WT-M (n = 10), **Scarb1^-/-^**-M (n = 5) and **Scarb1^-/-^**-M_AAVSOF_ (n = 9). Statistics are as described in **Figure 1** legend. Comparisons between male and female data within the same genotype or treatment gave the following significant differences between sexes: PL: WT F < M, *P* < 0.0001; TC: Scarb1^-/-^F> Scarb1^-/-^-M, *P* = 0.05; FC: Scarb1^-/-^F> Scarb1^-/-^-M *P* = 0.032; TG: WT F > M, *P* = 0.0101; mol% FC: WT F > M, *P* < 0.0001 and Scarb1^-/-^F> Scarb1^-/-^-M, *P* < 0.0001.


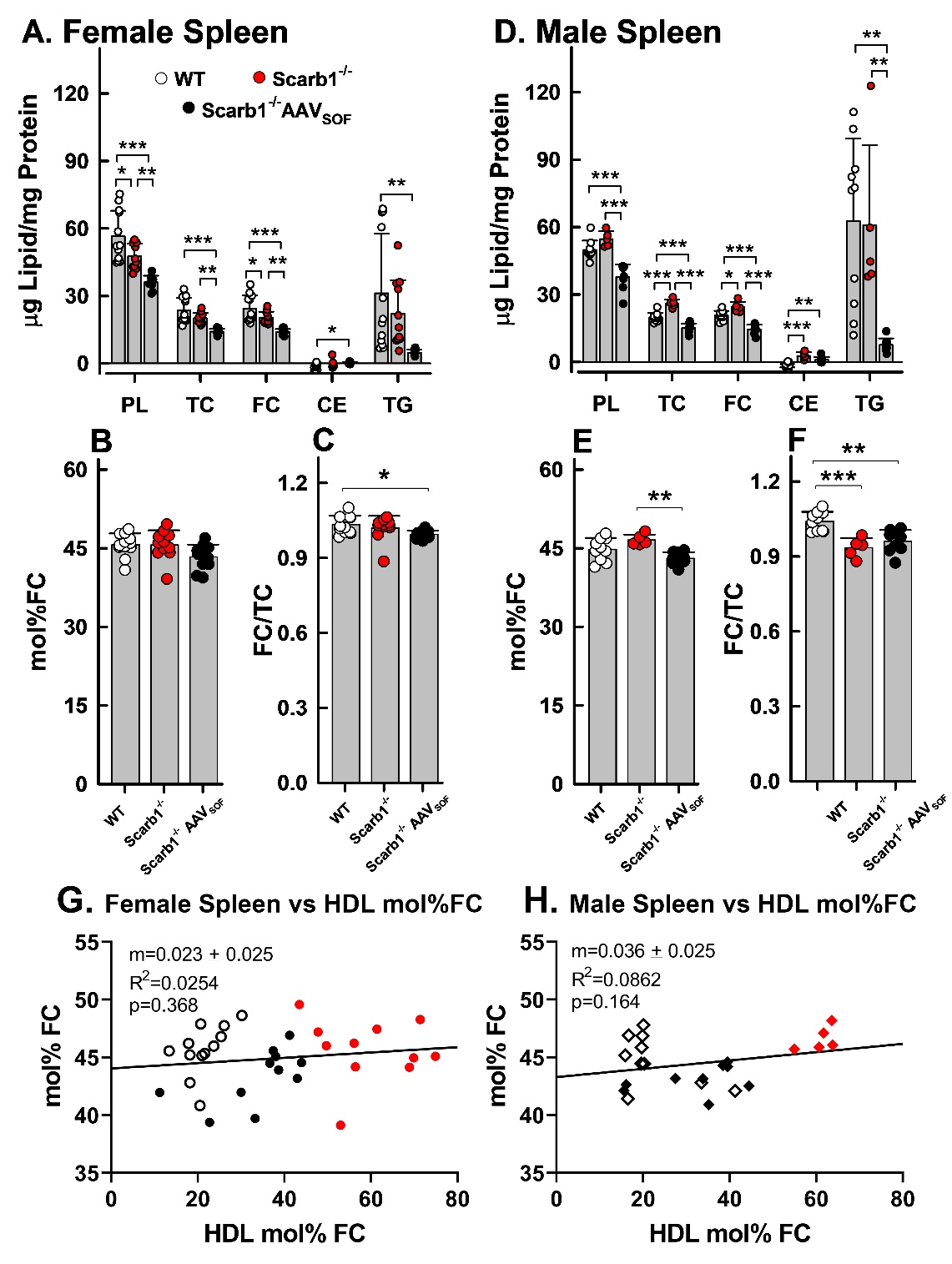


**Supplemental Fig. S6.** Spleen lipid composition. FC is decreased in spleens of female Scarb1^-/-^ mice, AAV_SOF_ decreases FC further, but mol% FC is the same in all three groups of female mice. FC is elevated in spleens of male Scarb1^-/-^ mice, AAV_SOF_ reduces spleen FC below WT levels, and decreases spleen mol %FC. **A-C and G:** Female**. D-F and H:** Male. The respective panels provide the lipid composition relative to protein (W/W), mol% FC, and the FC/TC ratio (W/W). **G, H**: Spleen-mol% FC does not correlate with HDL-mol% FC for either female or male mice. Data points are values for individual mice, and bars are mean + SD. Mice/group were: WT-F (n = 12), **Scarb1^-/-^**-F (n = 11), **Scarb1^-/-^**-F_AAVSOF_ (n = 11), WT-M (n = 10), **Scarb1^-/-^**-M (n = 5) and **Scarb1^-/-^**-M_AAVSOF_ (n = 9). Statistics are as described in **Figure 1** legend. Comparisons between male and female data within the same genotype or treatment showed no significant differences between male and female values.


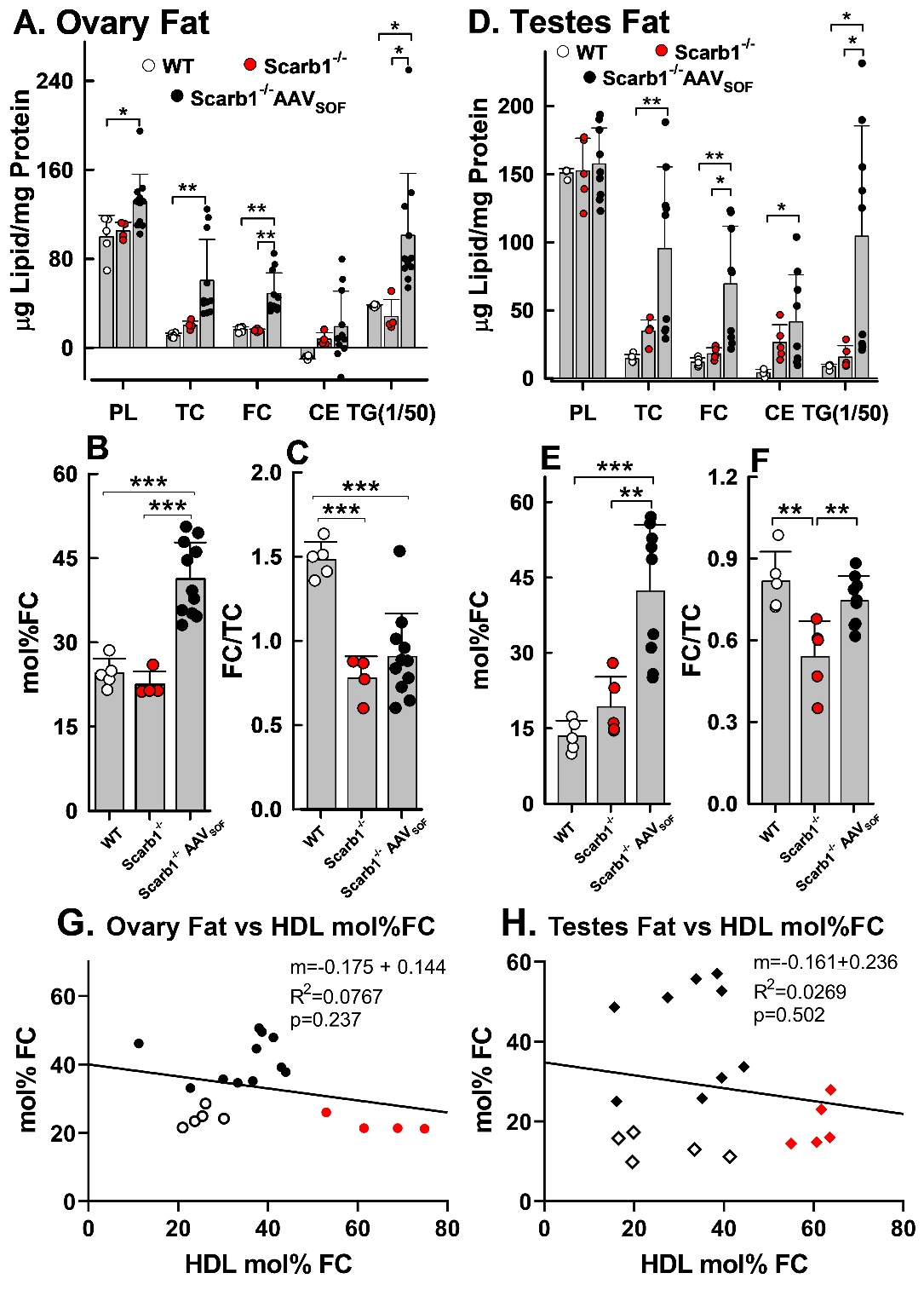


**Supplemental Fig. S7.** Ovary and testis fat lipid composition. FC, CE and mol% FC in Ovary Fat and Testis Fat of Scarb1^-/-^ mice are similar to levels in WT mice. AAV_SOF_ increases both FC and CE as well as mol %FC in these fat tissues. **A-C and G:** Ovary Fat**. D-F and H:** Testis Fat. The respective panels provide the lipid composition relative to protein (W/W), mol% FC, and the FC/TC ratio (W/W). **G, H**: Neither Ovary Fat mol % FC nor Testis Fat-mol% FC correlate with HDL mol %FC. Data points are values for individual mice, and bars are mean + SD. Mice/group were: Ovary Fat: WT-F (n = 5), **Scarb1^-/-^**-F (n = 4), **Scarb1^-/-^**-F_AAVSOF_ (n = 11); Testis Fat: WT-M (n = 5), **Scarb1^-/-^**-M (n = 5) and **Scarb1^-/-^**-M_AAVSOF_ (n = 9). Statistics are as described in Figure **1** legend.

| **Supplementary Table 1: Correlation of HDL-FC and Mol% FC with Tissue-FC and Mol% FC** | | | | | | | | |
| --- | --- | --- | --- | --- | --- | --- | --- | --- |
|  | Mol% /FC | | | | FC | | | |
|  | **Female** | | **Male** | | **Female** | | **Male** | |
|  | p | m | p | m | p | m | p | m |
| Plasma | <0.0001 | 0.647 ±0.067 | <0.0001 | 0.941 ± 0.049 | <0.0001 | 1.022 ± 0.04 | <0.0001 | 1.011 ± 0.060 |
| Erythrocytes | <0.0001 | 0.398 ± 0.079 | <0.0001 | 0.369 ± 0.081 | <0.0001 | 2.78 ± 0.40 | <0.0007 | 2.01 ± 0.523 |
| Lung | 0.004 | 0.138 ± 0.045 | 0.056 | 0.15 ± 0.075 | 0.0003 | 9.87 ± 2.43 | 0.004 | 10.22 ± 3.17 |
| Liver | 0.033 | 0.035 ± 0.016 | 0.380 | 0.027 ± 0.03 | 0.028 | 2.61 ± 1.13 | 0.005 | -1.88 ± 6.05 |
| Heart | 0.046 | 0.055 ± 0.027 | 0.048 | 0.057 ± 0.032 | 0.085 | 2.066 ± 1.16 | 0.032 | 2.414 ± 1.052 |
| Kidney | 0.046 | 0.055 ± 0.027 | 0.048 | 0.067 ± 0.032 | 0.129 | -1.55 ± 0.10 | 0.023 | -2.94 ± 1.20 |
| Adrenals | 0.124 | 0.139 ± 0.088 | 0.0026 | 0.328 ± 0.10 | 0.555 | 3.88 ± 6.5 | 0.47 | -6.92 ± 9.49 |
| Brain | 0.302 | -0.043 ± 0.041 | 0.624 | -0.027 ± 0.053 | 0.208 | -6.60 ± 5.13 | 0.745 | -2.10 ± 6.36 |
| Spleen | 0.368 | 0.023 ± 0.025 | 0.164 | -.036 ± 0.025 | 0.139 | -3.27 ± 2.15 | 0.083 | 3.06 ± 1.68 |
